# Physical health of patients under EIP - a service evaluation

**DOI:** 10.1192/bjo.2021.897

**Published:** 2021-06-18

**Authors:** Anu Priya, Keerthan Dhanasekar, James Hill-Cousins, Mudasar Aziz, Osama Suleiman, Katherine Mills, Megan Lloyd

**Affiliations:** 1 Bassetlaw Mental Health Services; 2 Sheffield University

## Abstract

**Aims:**

The aim of the project was to get a baseline of the number of patients who have had blood tests, ECG and physical health observations completed in the past 12 months.

**Method:**

There are 30 patients under Early Intervention in Psychosis team at Bassetlaw Hospital , Nottinghamshire. The elctronic notes and blood reporting system were checked for each of the patients, to get the data on blood test results , ECG reports and Physical health (Blood pressure, heart rate and weight) .

**Result:**

It was noted that 19/30 patients had Blood tests completed, 14/30 had ECG completed and 19/30 had physical health checks completed. All these patients except one were on antipsychotic medications.

**Conclusion:**

Further work is still required in getting 100% results for all these different variables. This may include the need to review the process of how we engage the patients for physical healthcare checks. With the inclusion of a physical healthcare worker now, we might be able to improve results. Hence this evaluation would be redone in a years' time.

